# Supplementary material for: Topographic structure and function of locus coeruleus noradrenaline neurons
Source: Nature. 2026 Sep 16;659(8140):459–68. doi: 10.1038/s41586-026-11026-0 (PMC13754599; doi:10.1038/s41586-026-11026-0)
Supplement: Supplementary file 1 [file 41586_2026_11026_MOESM1_ESM.pdf]

nature portfolio

https://doi.org/10.1038/s41586-026-11026-0

Supplementary information

Topographic structure and function of locus coeruleus noradrenaline neurons

In the format provided by the authors and unedited

Injection target (mice, cells)	Injection type	Injection volume	Coordinates from bregma (AP, ML, DV) (mm)
MOB (4, 928)	Pressure	150 nL per insertion	(4.28, 0.50, –2.30); (4.28, 1.20, –0.70)
CTX: PL, MO (5, 2,714)	Pressure	300 nL per insertion	(2.10, 0.33, –1.50); (1.70, 0.90, {–0.80, –0.40})
TH: VAL, VPM (7, 327)	Iontophoresis	5–7 min, 3 μA per insertion	(–0.82, 1.13, –3.28); (–1.82, 1.60, –3.30)
PAG (6, 1,546)	Pressure	200 nL	(–4.45, 0.40, –1.40)
CB: culmen, anisoform, paramedian (6, 3,829)	Pressure	200 nL, 300 nL	(–5.75, 0.00, –0.50); (–7.50, 2.00, {–1.75, –0.90})
SP: C4–C5 (7, 4,321)	Pressure	100 nL per insertion	(N.A., 0.35, –0.90)); (N.A., –0.35, –0.90))

Supplementary Table 1: Virus injections for retrograde tracing experiments. Summary of animal and cell counts by projection target and labelled soma location. Spinal cord injections were made bilaterally. All other injections were in the right hemisphere. Injection coordinates are relative to bregma (anterior-posterior, AP; medial-lateral, ML; dorsal-ventral, DV).

Injection target	Injection type	Injection volume	Coordinates from bregma (AP, ML, DV) (mm)
CTX: PL, MO	Pressure	300 nL per insertion	(2.10, 0.33, –1.50); (1.70, 0.90, {–0.80, –0.40})
TH: VAL, VPM	Pressure	300 nL per insertion	(–0.82, 1.13, –3.28); (–1.82, 1.60, –3.30)
CB: culmen, anisoform, paramedian	Pressure	200 nL, 300 nL	(–5.75, 0.00, –0.50); (–7.50, 2.00, {–1.75, –0.90})
SP: C4–C5	Pressure	100 nL per insertion	(N.A., 0.35, –0.90)); (N.A., –0.35, –0.90))

Supplementary Table 2: Virus injections for retro-seq experiments. Summary of animal and cell counts by projection target and labelled soma location. Spinal cord injections were made bilaterally. All other injections were in the right hemisphere. Injection coordinates are relative to bregma (anterior-posterior, AP; medial-lateral, ML; dorsal-ventral, DV).

Parameter	Prior distribution
$\alpha_{(-)}$	$[0, 1]$
$\alpha_{(+)}$	$[0, 1]$
α	$[0, 1]$
ζ	$[0, 1]$
β	$[0, 10]$
bias	$\mathcal{N}(0, 1)$

Supplementary Table 3: Reinforcement-learning model parameters.
