# Supplementary material for: Topographic structure and function of locus coeruleus noradrenaline neurons
Source: Nature. 2026 Sep 16;659(8140):459–68. doi: 10.1038/s41586-026-11026-0 (PMC13754599; doi:10.1038/s41586-026-11026-0)
Supplement: Supplementary file 2 [file 41586_2026_11026_MOESM2_ESM.pdf]

nature portfolio

Corresponding author(s): Jeremiah Y. Cohen, Jayaram V. Chandrashekar, Yoh Isogai

Last updated by author(s): Jul 20, 2026

Reporting Summary

Nature Portfolio wishes to improve the reproducibility of the work that we publish. This form provides structure for consistency and transparency in reporting. For further information on Nature Portfolio policies, see our Editorial Policies and the Editorial Policy Checklist.

Statistics

For all statistical analyses, confirm that the following items are present in the figure legend, table legend, main text, or Methods section.

n/a	Confirmed	
☐	☒	The exact sample size (*n*) for each experimental group/condition, given as a discrete number and unit of measurement
☐	☒	A statement on whether measurements were taken from distinct samples or whether the same sample was measured repeatedly
☐	☒	The statistical test(s) used AND whether they are one- or two-sided<br>*Only common tests should be described solely by name; describe more complex techniques in the Methods section.*
☐	☒	A description of all covariates tested
☐	☒	A description of any assumptions or corrections, such as tests of normality and adjustment for multiple comparisons
☐	☒	A full description of the statistical parameters including central tendency (e.g. means) or other basic estimates (e.g. regression coefficient) AND variation (e.g. standard deviation) or associated estimates of uncertainty (e.g. confidence intervals)
☐	☒	For null hypothesis testing, the test statistic (e.g. *F*, *t*, *r*) with confidence intervals, effect sizes, degrees of freedom and *P* value noted<br>*Give P values as exact values whenever suitable.*
☐	☒	For Bayesian analysis, information on the choice of priors and Markov chain Monte Carlo settings
☐	☒	For hierarchical and complex designs, identification of the appropriate level for tests and full reporting of outcomes
☐	☒	Estimates of effect sizes (e.g. Cohen's *d*, Pearson's *r*), indicating how they were calculated

Our web collection on statistics for biologists contains articles on many of the points above.

Software and code

Policy information about availability of computer code

Data collection	Neuralynx (Cheetah 5.7.4), Open Ephys 1.0.0, Arduino 1.6.9 to 2.0.4, Bonsai 2.8, ThorCam 3.7.0, Python 3.8 to 3.14, ZEISS ZEN 3.14, ExA-SPIM control (https://github.com/AllenNeuralDynamics/exaspim-control), NIS-Elements 5.3004.1553.0, µManager 2.0.3 20240306, SpinView 1.29.0.5
Data analysis	Python 3.8 to Python 3.14, R 4.6.1, PyStan 3.10.0, MATLAB 2026a, HortaCloud 9.24.1, Neuroglancer 2.34, IBL ephys alignment gui (https://github.com/AllenNeuralDynamics/ibl-ephys-alignment-gui), QuickNII (https://www.nitrc.org/projects/quicknii), VisuAlign (https://www.nitrc.org/projects/visualign/), Cellpose

For manuscripts utilizing custom algorithms or software that are central to the research but not yet described in published literature, software must be made available to editors and reviewers. We strongly encourage code deposition in a community repository (e.g. GitHub). See the Nature Portfolio guidelines for submitting code & software for further information.

Data

Policy information about availability of data

All manuscripts must include a data availability statement. This statement should provide the following information, where applicable:

- Accession codes, unique identifiers, or web links for publicly available datasets
- A description of any restrictions on data availability
- For clinical datasets or third party data, please ensure that the statement adheres to our policy

Data are available at https://github.com/AllenNeuralDynamics/locus_coeruleus_norepinephrine_neurons, https://assets.nemoarchive.org/col-p9d5w39, and https://codeocean.allenneuraldynamics.org/collections/9cf044ce-93c7-4c7e-bfa1-5d8c37aa42ec

Research involving human participants, their data, or biological material

Policy information about studies with human participants or human data. See also policy information about sex, gender (identity/presentation), and sexual orientation and race, ethnicity and racism.

Reporting on sex and gender	N/A
Reporting on race, ethnicity, or other socially relevant groupings	N/A
Population characteristics	N/A
Recruitment	N/A
Ethics oversight	N/A

Note that full information on the approval of the study protocol must also be provided in the manuscript.

Field-specific reporting

Please select the one below that is the best fit for your research. If you are not sure, read the appropriate sections before making your selection.

☒ Life sciences ☐ Behavioural & social sciences ☐ Ecological, evolutionary & environmental sciences

For a reference copy of the document with all sections, see nature.com/documents/nr-reporting-summary-flat.pdf

Life sciences study design

All studies must disclose on these points even when the disclosure is negative.

Sample size	No predetermined sample size. Sample sizes chosen based on field standards.
Data exclusions	No data exclusion.
Replication	Results reproduced across mice (varying sample sizes across experiments).
Randomization	Mice randomly allocated into experimental groups.
Blinding	Investigators blind to group allocation.

Reporting for specific materials, systems and methods

We require information from authors about some types of materials, experimental systems and methods used in many studies. Here, indicate whether each material, system or method listed is relevant to your study. If you are not sure if a list item applies to your research, read the appropriate section before selecting a response.

Materials & experimental systems

n/a	Involved in the study
☐	☒ Antibodies
☒	☐ Eukaryotic cell lines
☒	☐ Palaeontology and archaeology
☐	☒ Animals and other organisms
☒	☐ Clinical data
☒	☐ Dual use research of concern
☒	☐ Plants

Methods

n/a	Involved in the study
☒	☐ ChIP-seq
☒	☐ Flow cytometry
☒	☐ MRI-based neuroimaging

Antibodies

Antibodies used

rabbit anti-TH (ab152, Millipore; 1:500), goat anti-rabbit (A-11012, Alexa 594, Life Technologies; 1:1000), goat anti-chicken (A-11039, Alexa 488, Life Technologies), donkey anti-rabbit (A-21206, Alexa 488, Life Technologies; 1:1000), Rabbit anti-GFP (ab290, Abcam), Goat anti-tdTomato (AB8181, SICGEN)

Validation

Validation of antibodies are available on manufacturer's websites:

ab152: "By western blot, AB152 selective labels a single band at approximately 62kDa (reduced) corresponding to Tyrosine Hydroxylase. It is expected that the antibody will react with most mammalian and many non-mammalian species."

A-11012: "To minimize cross-reactivity, these goat anti-rabbit IgG whole antibodies have been cross-adsorbed against human IgG, human serum, mouse IgG, mouse serum, and bovine serum. Cross-adsorption or pre-adsorption is a purification step to increase specificity of the antibody resulting in higher sensitivity and less background staining. The secondary antibody solution is passed through a column matrix containing immobilized serum proteins from potentially cross-reactive species. Only the nonspecific-binding secondary antibodies are captured in the column, and the highly specific secondaries flow through."

A-11039: "Anti-Chicken secondary antibodies are affinity-purified antibodies with well-characterized specificity for the chicken immunoglobulins and are useful in the detection, sorting or purification of its specified target."

A-21206: "To minimize cross-reactivity, these donkey anti-rabbit IgG whole antibodies have been affinity-purified and show minimum cross-reactivity to bovine, chicken, goat, guinea pig, hamster, horse, human, mouse, rat, and sheep serum proteins. Cross-adsorption or pre-adsorption is a purification step to increase specificity of the antibody resulting in higher sensitivity and less background staining. The secondary antibody solution is passed through a column matrix containing immobilized serum proteins from potentially cross-reactive species. Only the nonspecific-binding secondary antibodies are captured in the column, and the highly specific secondaries flow through."

ab290: "Anti-GFP antibody (ab290) is a rabbit polyclonal antibody and is validated for use in ELISA, EM, ICC/IF, IHC-FoFr, IHC-Fr, IHC-FrFl, IHC-P, IP and WB in human samples.
Anti-GFP antibody (ab290) was first used in a scientific publication in 2002 and has been cited over 3370 times in peer reviewed journals. It's performance in Western blot, immunofluorescence and IHC in human and mouse samples is trusted by the scientific community.
GFP antibodies are used to visualize proteins labelled with this tag in a variety of applications (for example imaging and Flow cytometry). To enable specific detection of your tagged protein, Anti-GFP antibody (ab290) has been validated in ELISA, Western Blot and imaging applications.
Anti-GFP antibody (ab290) specifically detects GFP (UniProt ID: P42212; Molecular weight: 27kDa) and is sold in a convenient trial size to enable initial testing (50 µL) and larger sizes for subsequent scaling up experiments (500 µL)."

ab8181: "In 293HEK cells transfected with cds plasmid detects a band of 55 kDa by Western blot. It also detects tdTomato in brain sections by IHC. This antibody (AB8181) is specific for tdTomato and mCherry proteins. It does not cross-react to GFP (green fluorescent protein)."

Animals and other research organisms

Policy information about studies involving animals; ARRIVE guidelines recommended for reporting animal research, and Sex and Gender in Research

Laboratory animals

Dbh-cre mice (The Jackson Laboratory, 033951), C57BL/6J mice (The Jackson Laboratory, 000664), Ai65 mice (The Jackson Laboratory, 021875), RCFL-H2B-GFP mice (Jackson Laboratory, 028581), ranging from P28 to P112.

Wild animals

No wild animals used.

Reporting on sex

Mice of both sexes were included.

Field-collected samples

No field-collected samples.

Ethics oversight

All surgical and experimental procedures were in accordance with the National Institutes of Health Guide for the Care and Use of

Ethics oversight	Laboratory Animals and approved by the Allen Institute Animal Care and Use Committee or Johns Hopkins Animal Care and Use Committee.

Note that full information on the approval of the study protocol must also be provided in the manuscript.

Plants

Seed stocks	N/A
Novel plant genotypes	N/A
Authentication	N/A
