# Supplementary material for: Topographic structure and function of locus coeruleus noradrenaline neurons
Source: Nature. 2026 Sep 16;659(8140):459–68. doi: 10.1038/s41586-026-11026-0 (PMC13754599; doi:10.1038/s41586-026-11026-0)
Supplement: Supplementary file 3 [file 41586_2026_11026_MOESM3_ESM.pdf]

nature portfolio

Peer Review File

Topographic structure and function of locus coeruleus norepinephrine neurons

Corresponding Author: Dr Jeremiah Cohen

Any redactions in this file are there to maintain patient confidentiality, the confidentiality of unpublished data, or to remove third-party material.

This file contains all reviewer reports in order by version, followed by all author rebuttals in order by version.

Attachments originally included by the reviewers as part of their assessment can be found at the end of this file.

Version 0:

Reviewer comments:

Referee #1

(Remarks to the Author)
Su and Cohen present a very clear, well written, and novel report about the diversity of locus coeruleus (LC) norepinephrine (NE) neurons, and their functions in reinforcement learning.

LC NE neurons were found to cluster to two types based on their firing properties and spike shapes, and then it was determined that these clusters support distinct functions of the LC in learning and behavioral control.

I believe this paper fits well within Nature, because it represents a simple and clear story that will have a lasting impact on the field.

That said, I will ask the authors to revise and modify their paper to make their results clearer and increase the impact of the data.

(1) All models have assumptions, and none are correct. In a simple task, many RL agents will match behavior, but their internal workings may differ from how the animal's brain, and from each other (model vs. model). The authors know this well, and accordingly use Extended Figs to argue for their approach: because the model choices mostly match the mouse (panel H) and due to other nice analyses performed by the authors (e.g., licking magnitude following big negative reward prediction errors derived by the model shows that mice indeed strongly expected a reward). As predicted by my earlier comments, I think the latter approach is particularly important and I am glad the authors did this. Nonetheless, unless the authors can address how the mice estimate RPEs and values (e.g., how close to the model versus not),
I would address this set of issues in a few sentences in the Discussion. I suspect many different models would give identically good fits in this task. I think this is critical for future studies comparing Type1 and Type2 responses to model-derived RPEs, and for comparing across studies that use diff models deriving RPEs in LC and other modulators.

Related to this, I also have a few specific suggestions and questions for the authors.

-- Figure 1F – authors state that the change in choice likelihood is monotonically increasing as a function of model-derived RPE. It seems rather a steep function, largely driven by particularly large + RPEs. Please explore this a bit more. Also, The activity of LC Type-1 neurons seems to really grow with model derived RPE very finely. Perhaps, the behavior has additional sources of variability beyond RPE? E.g., if later in the session animals stop weighting small RPEs similarly as big, maybe there is behavioral distortion ?

-- The licking following no-reward trials is a great analysis the authors use to show the mouse is tracking value like the model. Is there a relationship of activity and behavior on even a finer timescale? In other words, how good is trial-by-trial LC activity at predicting behavior (e.g., switching, RTs). This is a tough analysis for any brain area to "pass" and if the authors fail in finding a relationship of course it does not mean LC is not 'doing RPEs' for learning, but if the authors succeed, it

would be very interesting to report (albeit negative findings in this type of analysis would be difficult to fully interpret).

-- Motor versus. cognitive aspects of LC. What is the relationship of LC and licking state? I think it's clear that LC is not just a motor signal but would be great to have a sentence or two with stats saying it has no relationship to licking at baseline. Similarly, what is the relationship of pupils and LC activity (e.g., at baseline)? And, relationship of pupil vs RPE? Does perturbation of LC modulate pupils as strongly during any epoch of the task, or most strongly during outcome?

(2) The lack of bidirectionality in the LC RPE of the Type 1 cells should be thought about in this report. In the average it appears there is a little activation for all RPEs (positive and negative; Figure 3D). This type of activity that is correlated with RPE may have different functions from a bidirectional VTA dopamine RPE. I would like the authors to discuss this in the context of their perturbation, modeling, task, and relationship to other modulators. This issue may also become important as investigators study the functions of Type1 and Type 2 neurons in future studies using aversive stimuli, and in considering how Type 1 cells modulate cortex (e.g., gain versus something else).

(3) Type 2 neurons did not encode quantitative model derived RPE but did have a strong bidirectional discrimination of + and – RPEs. I found that result very impressive and surprising: as if they are categorically parsing good and bad surprises (even in a bidirectional manner, unlike Type 1). This type of signal could be used in foraging to decide when to go versus stay and for other purposes. I would recommend the authors to discuss this signal more in the Discussion and importantly to acknowledge that Type 2 lack of negative RPE coding maybe due to some complex process such as how they subjectively weight different RPEs or other algorithm differences between Type 1 and Type 2 cells.

Also, were Type 2 cells inhibited by Go-cue? Did the Type-1 excitation to go cue have anything to do with trial behavior?

Minor points

Maximum magnitude of licking is used to show the degree to which the mice believed they would get a reward in no-reward trials. Why not use licking-persistence also: the duration of significantly elevated licking. This approach works for neural activity (see Zhang et al., 2019) and may work here too.

Finally, if it was me, I would make some index or measure of behavioral-prediction and compare Type 1 and Type 2 neurons. E.g., how good are switch predictions (or RT predictions in next trial) based on current trial outcome related activity of Type 1 versus Type 2 neurons. After all the complex analyses, that would show readers something simple to "take-away".

Dayan and Yu, 2006 is cited for determining LC functions in the introduction. I would highlight their contribution by having a separate sentence for biological data references versus modeling references, so that their contribution (modeling) can be appreciated, and so that the readers that are not familiar with the literature can easily understand which previous work was experimental and which was theoretical.

Along those lines, I would recommend having a few sentences in Discussion dedicated to comparing LC results to SNc/VTA and basal forebrain. I think that would again highlight the importance and uniqueness of the current work.

Referee #2

(Remarks to the Author)
The manuscript by Su & Cohen attempts to address an important question in the field relating to how norepinephrine release in the cortex influences learning, and whether modularity exists at the level of the LC which may affect trial-by-trial learning. This is currently an actively debated topic with respect to the LC, it's larger roles in behavior, and the actions of norepinephrine generally. So the authors do test a novel, important and impactful series of hypotheses in this study. The studies is rigorously designed, modeled, and conducted overall, however there are concerns about the strength of the conclusions as it currently stands.

Using optogenetic tagging of norepinephrine-producing LC neurons (Dbh+ LC-NE neurons), Su & Cohen record from many LC-NE neurons (149 neurons from 4 mice) simultaneously in an awake behaving mouse performing a complex reinforcement learning task. Consistent with previous work, they identify two main classes of LC-NE neurons based on their electrophysiological properties, type I neurons, characterized by a wide action potential shape, and type II neurons with thinner action potential waveforms. While these classes were known, examining their role in reinforcement learning is novel and quite interesting a finding. The authors demonstrate differences between the two classes and reinforcement learning measures, primarily that the response of type I neurons is graded and scales with RPE, while type II LC-NE neurons are excited when reward is expected but not delivered (negative RPE). These findings are novel and extremely interesting, and warrant deeper analysis. Unfortunately, the subsequent functional experiments manipulating type I neurons optogenetically and assessing the activity of LC-NE projections during reinforcement learning suffer from numerous issues leaving their interpretation challenging, and tempering enthusiasm for the manuscript as a whole. The authors' claims about LC-NE influencing RPE can be further strengthened and the noradrenergic RPE signal at the frontal cortex can be further clarified.

Major comments:
1) The authors use optogenetic inhibition of LC-NE neurons via the excitatory opsin GtACR2, and attempt to preferentially

inhibit type I neurons, which should reduce RPE on stimulation trials and decrease the value of the selected action. However, no evidence for preferential targeting of type I neurons is really presented. This is somewhat problematic because the opposite prediction would be made if type II neurons are inhibited. While the authors state "low" irradiance is used (still in the mW range), there is no data nor histology presented to fully support this contention. The authors may wish to try strategies using tapered or fibers/LEDs that direct light at an angle, or potentially present histology demonstrating bleaching selectively of the fluorophore in dorsal LC-NE neurons. As it stands, given that type II neurons are 1) more prevalent overall in the LC than type I neurons (86 out of 149 LC-NE neurons were type II), 2) have higher baseline firing rates than type I neurons (Extended Data Figure 2c), and that the only histological image of GtACR2 expression shows high expression in both dorsal and ventral LC (Figure 4a), it is hard to justify a hypothesis and subsequent predictions based on manipulation of type I neurons only. This is the biggest concern related to the conclusions of the paper. If this manipulation is the effect of inhibiting both type I and type II neurons, the relevance of their RPE-specific activity patterns becomes unfortunately more tenuous and less novel.

2) A similar question exists with the following experiment using fiber photometry to examine LC-NE neuron activity as well as the activity of LC-NE terminals in the medial PFC. At the somatic level, it is unclear why photometry signals preferentially resemble type I and not type II neurons. Again, a careful attempt to quantify and present histological targeting of the fibers within the LC would be very useful. If fibers were dorsal, a strong type I signal might be expected. Given the extremely low N in this experiment (n = 2), there is much to be gained from recording additional mice and correlating signals with location of the photometry fiber in the D/V axis. If a photometry signal obtained in very ventral LC (where type II neurons are prominent) does not resemble the convolved signal found in Figure 5c, then interpreting a lack of this signal in the PrL may be more spurious. Overall, more experiments in this case are needed to draw firmer conclusions about the nature of the signal in both LC and PrL. The authors have the ability to do this, so it is more about adding N's than technically challenging an approach.

3) In the silencing of type I LC-NE neurons, the authors predicted that it will lead to artificially reducing RPE, and therefore decreasing the value of the chosen action and increasing the P(switch). Yet, in Fig 3f, the authors showed that "Higher activity of type I neurons correlated with larger change in choice likelihood". Thus, it would be particularly helpful if the authors can clarify the discrepancy between their observation and the model prediction. As stated above a weakness in the silencing experiment is the assumption that type I neurons are preferentially activated using the low irradiance by targeting the more dorsal neurons. From Fig 2f, the type II neurons appear to be ~20% of the neurons in the dorsal layer, thus, their participation in RPE cannot be discounted, especially with relatively high correlation of choice switching at its lower firing range (Fig 3f). What would incorporating predictions of type II neuron silencing look like? Does the degree of silencing as captured by the change in pupil diameter (Fig 4d) correlate with the probability of switching (Fig 4f)? These are important considerations and more experiments are needed to firm the conclusions at the later end of the manuscript in this regard. If possible to complete the novelty and impact of the findings are most certainly very exciting.

4) Also, with the GtACR2 silencing, the protocol uses a 2s constant stimulation followed by a 2s ramp down to minimize post-inhibitory rebound excitation, but from the changes in pupil diameter (Fig 4c), the increase right after stopping the laser suggests that a rebound depolarization may still be present and represented in the activity. Furthermore, per the methods section, the trial duration for silencing experiment is 3.0s with a minimum of 0.1s inter-trial intervals, it is unclear whether the silencing duration of 4s is bleeding into the initiation of a new trial. A bit more characterization of the inhibition parameters might be helpful. Perhaps the authors can do a few slice experiments to characterize these parameters, to guide the in vivo work. Of course they aren't totally equivalent, but it might make the other concerns above easier to address if there is a better set of inhibition parameters defined. Alternatively, the authors could label the dorsal neurons via a retro-CAV approach

5) Additionally, given the approximate linear relationship between type I neurons and RPE and choice switching, it would further strengthen the authors claim if they also preferentially activated the type I neurons to determine whether their model predictions will be correct.

6) In correlating LC-NE activity in the PrL to the RPE, the authors saw that the linear relationship holds at positive RPE, but does not change at negative RPE, and attributed the lack of positive correlation at negative RPE to a difference in measurement technique. While fiber photometry may be less sensitive at axonal terminals, it would be informative to have LC-NE calcium activity measured by fiber photometry at the soma to see if this may indeed be the explanation. Moreover, a measurement of NE in the PrL during the reinforcement learning assay would lend further support to the specific role of NE in signaling RPE.

Minor comments:
-The methods state that 7 mice were used for GtACR2 and control experiments, but the data plotted in Figure 4 make it appear that there are more mice in each group. Perhaps I was confused about how this was conducted.
-It is unclear if GtACR2-expression itself is affecting mice behavior, as the mice cohort overall appears to have slower response time than the EYFP cohort (Extended fig 4b) regardless of laser use, and had much higher lick rate in response to reward regardless of laser use (Extended fig 4c).

Referee #3

(Remarks to the Author)
Su Z and Cohen JY, Two types of locus coeruleus norepinephrine neurons drive reinforcement learning.

The authors report on a study in which they identified, based on waveform shape and anatomical location, two types of NE neurons. They then examined the responses of these neurons, as well as the effects of silencing these populations on choices in a restless bandit RL task. They found that the more dorsal population of type I neurons coded a positive RPE, whereas the more ventral population of type II neurons coded lack of reward. Preferentially silencing type II neurons led to an increase in switch probability following a lack of reward.

This is a nicely done study with some novel findings. The behavioral is well-controlled, and the authors convincingly show differences in the cell populations with respect to response properties. The opto inhibition experiments are also interesting.

I have two primary comments on this manuscript. First, with respect to the dopamine vs. norepinephrine innervation of cortex, it is not so clear that they differ that much. Perhaps there are clear differences in the rodent in the dopamine vs. norepinephrine innervation of cortex, but I’m not aware of studies showing this. Also, there is not much norepinephrine in the anterior striatum, but I believe there is more in the caudal striatum, and it’s possible that the caudal striatum is mediating these effects.

Second, while the experiments nicely show differences in the directional licking task, there are now numerous papers showing that, while the responses of dopamine neurons and the effects of their manipulation in similar tasks might be clear, the functional role of dopamine neurons is actually far more complex. Thus, the results in this simple experiment on NE are highly interpretable. But it seems that subsequent experiments with more complex paradigms would likely lead to different interpretations.

In sum, while this is a nice study, I think a more convincing case would require the use of multiple paradigms looking at factors like sensory preconditioning, outcome type switching, perhaps motor behavior, the explore-exploit tradeoff (and arguably other factors too numerous to list), along with a direct comparison of the effects of dopamine manipulations in matched experiments. This, combined with a clear anatomical study of projection patterns of dopamine vs. norepinephrine, and maybe information on which targets are most relevant for dopamine vs. norepinephrine in particular tasks.

Version 1:

Reviewer comments:

Referee #1

(Remarks to the Author)
Thank you for addressing all of my questions and concerns. This is a remarkable and elegant piece of work.

(Remarks on code availability)
n/a

Referee #2

(Remarks to the Author)
In this substantially revised manuscript from the Cohen group and Allen Institute, the authors have now provided one of the first and most comprehensive data sets on LC heterogeneity across both molecular and anatomical domains, which is then further bolstered by behavioral data and electrophysiology demonstrating functional separation between the dorsal and ventral LC populations with meaningful implications for understanding the LC-NE system in learning and other behavioral domains. The paper is a tour - de - force, and will become the new reference atlas for LC circuits going forward. I only have a few minor corrections to suggestion before the MS is acceptable for publication.

Minor comments:

1. The main figures of the paper tell a very strong narrative regarding the anatomical separation of LC, across various domains, but what is sort of missing, but buried in the supplement is the molecular profiling and BARseq data. It would be useful to remove some of the anatomy, or simply add a few panels which include more of the merFISH or BARseq data in the main manuscript.

2. On a related note, it is difficult in the current form to access the seq data in terms of looking at other expression of other genes not currently shown in the figures or analysis? It would be useful to see data sets from the seq data that compare to smaller data sets and the Allen Cell Atlas in terms of expression profiles for genes, know to have -cre driver line access, so that the field can potentially independently synthesize combinations of expression patterns with cre- and/or flp lines for accessing some of the unique LC projection modules highlighted here. Maybe I missed where that data is, but since there are 3 other LC seq data sets, some comparisons or mention to some of the genes IDed in those data sets, or the lack thereof, could be useful for integrating this nice body of work with that larger field. For me, this is simply show more plots (dot plots or other they have used) for other genes in the LC which are relevant. The data as presented in MERFISH (it isn't clear if this was a custom run, or a run only using the baseline MERFISH gene set), so it would just integrate this new work more into that existing framework. Albeit, the other data sets have a lot less read depth and cells, but since many of the populations overlap here, I think a little more effort to share more of that data is warranted in the final main figures of the MS. S9 and S10 contain some of this information, but there is pharmacological evidence that there are differences in neuronal responses to

various receptor ligands in LC, and so a little more of what was looked for in that domain could be presented in some form, unless those genes weren't assessed by the MERFISH.

3.

(Remarks on code availability)

Referee #3

(Remarks to the Author)
I would like to thank the authors for revising the manuscript and replying to my initial comments. The study provides descriptive information about the anatomy of the LC and its projections, and the activity of the neurons in one task. While I appreciate that the authors would like to limit the scope of their paper, I am not sure that I find that the results provide a clear understanding of either the anatomy or the function of the LC. Furthermore, a contrast with other systems would be very useful, but it is not provided.

(Remarks on code availability)

Open Access This Peer Review File is licensed under a Creative Commons Attribution 4.0 International License, which permits use, sharing, adaptation, distribution and reproduction in any medium or format, as long as you give appropriate credit to the original author(s) and the source, provide a link to the Creative Commons license, and indicate if changes were made.
In cases where reviewers are anonymous, credit should be given to 'Anonymous Referee' and the source.
The images or other third party material in this Peer Review File are included in the article’s Creative Commons license, unless indicated otherwise in a credit line to the material. If material is not included in the article’s Creative Commons license and your intended use is not permitted by statutory regulation or exceeds the permitted use, you will need to obtain permission directly from the copyright holder.
To view a copy of this license, visit https://creativecommons.org/licenses/by/4.0/

Jeremiah Y. Cohen, Ph.D.
Allen Institute for Neural Dynamics
Seattle, WA

April 14, 2026

Dear reviewers,

We appreciate your enthusiasm and feedback on our previous manuscript. In the course of our new experiments, we discovered deep links between the structure and function of locus coeruleus (LC) norepinephrine (NE) neurons. Our revised manuscript, "Topographic structure and function of locus coeruleus norepinephrine neurons," therefore reports on the spatial organization of LC-NE neuronal morphologies, gene expression, and activity during behavior. The latter results expanded from the initial discovery of reward-related activity to activity correlated with choice as well.

We found that NE neurons are organized with exquisite topography in the LC. Our major findings are summarized below. Note that the first two are new, and the third is a significant expansion of what was reported in the original submission.

1. **Axonal projections of individual LC-NE neurons are spatially organized**
   Reviewers asked about the anatomical organization of LC and the activity we measured. The literature currently has no information on the projections of individual LC-NE neurons. Thus, we reconstructed the complete morphologies of 130 LC-NE neurons. As a group, LC-NE projections covered most of the brain (except for minor projections to the striatum) and spinal cord. However, individual neurons had preferred targets and were organized according to the dorsal-ventral locations of the cell bodies. We compared these results to additional experiments using sequencing-based projection analysis (MAPseq/BARseq) and retrograde tracing. These results are presented in Figures 1 and 2 and their supplements, and are the foundation of the spatial analyses in subsequent figures.

2. **LC-NE neurons have graded gene expression in space that maps onto the projections**
   Reviewers asked about the spike waveforms, particularly with regard to space and projection targets. We wondered whether LC-NE neurons are made up of discrete cell types. Thus, we studied their transcripts using single-nucleus RNA sequencing and found that LC-NE neurons comprise a single population with graded gene expression. We mapped this gradient to spatial coordinates using a spatial transcriptomics dataset, finding that it aligned with variation in projection targets. These results are presented in Figure 3 and its supplements.

3. **Choice- and reward-related activity of LC-NE neurons is spatially organized, aligning with projections and gene expression**
   Reviewers asked about the spatial organization of task-related activity and the interpretation of observed correlations. We doubled the dataset of single LC-NE neuron activity while mice performed a dynamic task, learning from the outcomes of their actions. In these new experiments, we reconstructed the locations of recorded cells and identified some with projections to cortex using antidromic stimulation and collision tests.

Dorsal LC-NE neurons with projections to the cerebral cortex were excited when the mouse’s choice differed from its previous one and when the mouse received a reward that was better than expected (i.e., reward prediction error, RPE). When background activity of ventral LC-NE neurons was high, mice tended to ignore go cues indicating potential reward availability. These results are presented in Figures 4-6 and their supplements.

We believe the manuscript is substantially improved and now reports foundational discoveries about LC-NE neurons. Responses to each point are below (reviewer comments in italics).

In sum, our studies reveal a link between the brain-wide organization of a neurotransmitter system and its functions in behavior. We are eager to hear your thoughts.

For the authors,

Jeremiah Y. Cohen, Ph.D.

Reviewer 1 (R1)

We thank R1 for their enthusiasm and suggestions for improvement. There were three main points. The first was about the assumptions of the models of behavior, the second was about the results of the perturbation experiments, and the third was about the relationship between activity of LC-NE neurons excited by lack of reward and behavior.

> *(1) All models have assumptions, and none are correct. In a simple task, many RL agents will match behavior, but their internal workings may differ from how the animal's brain, and from each other (model vs. model). The authors know this well, and accordingly use Extended Figs to argue for their approach: because the model choices mostly match the mouse (panel H) and due to other nice analyses performed by the authors (e.g., licking magnitude following big negative reward prediction errors derived by the model shows that mice indeed strongly expected a reward). As predicted by my earlier comments, I think the latter approach is particularly important and I am glad the authors did this. Nonetheless, unless the authors can address how the mice estimate RPEs and values (e.g., how close to the model versus not),*
> *I would address this set of issues in a few sentences in the Discussion. I suspect many different models would give identically good fits in this task. I think this is critical for future studies comparing Type1 and Type2 responses to model-derived RPEs, and for comparing across studies that use diff models deriving RPEs in LC and other modulators.*

We agree that each model comes with assumptions, none fully capturing the animal's strategy, even in a simple RL task. We revised the language in the Results and rewrote the Discussion. We compared the fit of the *Q*-learning model to other models. As R1 predicted, many RL models do similarly well in capturing overall behavior. For parsimony, we report on one RL model, with additional metrics to quantify goodness of fit (see Methods). Two new regressions, capturing different aspects of behavior, are also presented in Figures 5a and 5e. This is an important addition, as we now show the relationship between LC-NE activity and overall task engagement, as well as switch vs. stay choices (more on this below). Using new data with Neuropixels probes and new approaches for localizing the recorded neurons in space with high accuracy, we found a spatial relationship in this analysis (Figure 5). We tested this with new experiments to record from LC-NE neurons with projections to cortex (Figures 5 and 6).

> *Related to this, I also have a few specific suggestions and questions for the authors.*
>
> *-- Figure 1F – authors state that the change in choice likelihood is monotonically increasing as a function of model-derived RPE. It seems rather a steep function, largely driven by particularly large + RPEs. Please explore this a bit more. Also, The activity of LC Type-1 neurons seems to really grow with model derived RPE very finely. Perhaps, the behavior has additional sources of variability beyond RPE? E.g., if later in the session animals stop weighting small RPEs similarly as big, maybe there is behavioral distortion ?*

Thanks for this suggestion. We developed a new analysis linking changes in behavior to RPE (Figure 6c), showing finer changes in choice behavior when analyzed this way:

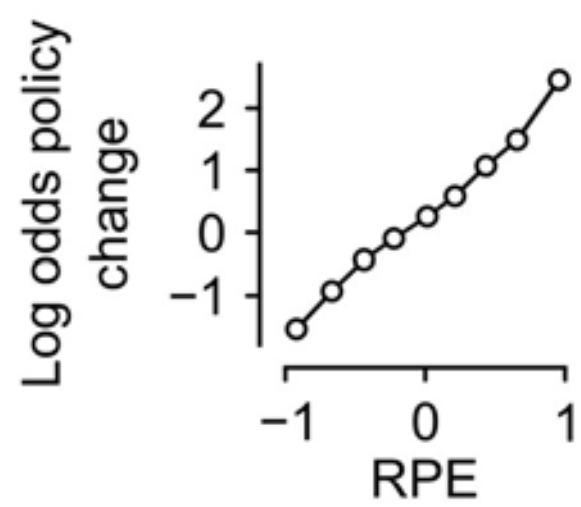

-- The licking following no-reward trials is a great analysis the authors use to show the mouse is tracking value like the model. Is there a relationship of activity and behavior on even a finer timescale? In other words, how good is trial-by-trial LC activity at predicting behavior (e.g., switching, RTs). This is a tough analysis for any brain area to "pass" and if the authors fail in finding a relationship of course it does not mean LC is not 'doing RPEs' for learning, but if the authors succeed, it would be very interesting to report (albeit negative findings in this type of analysis would be difficult to fully interpret).

Thanks for this question. It led to a new discovery. We analyzed activity on switch (i.e., different choice from the previous one) vs. stay (i.e., same choice as the previous one) choices. We doubled the sample size of single units and reconstructed their precise locations in LC. We found a spatial relationship: dorsal cells tended to have higher activity when mice switched choices, whereas ventral cells tended to have higher activity when mice stayed with the prior choice (Figure 5). Our new discoveries of the anatomical organization of LC-NE neurons (see Figures 1, 2, and their supplements) revealed that dorsal neurons project to rostral regions, including the cortex. Thus, we hypothesized that LC-NE input to the cortex would be more active during switch vs. stay choices. This was indeed the case, both at the single cell level (using antidromic stimulation with collision tests to identify them) and bulk axonal activity in frontal cortex (Figures 5j and 5k):

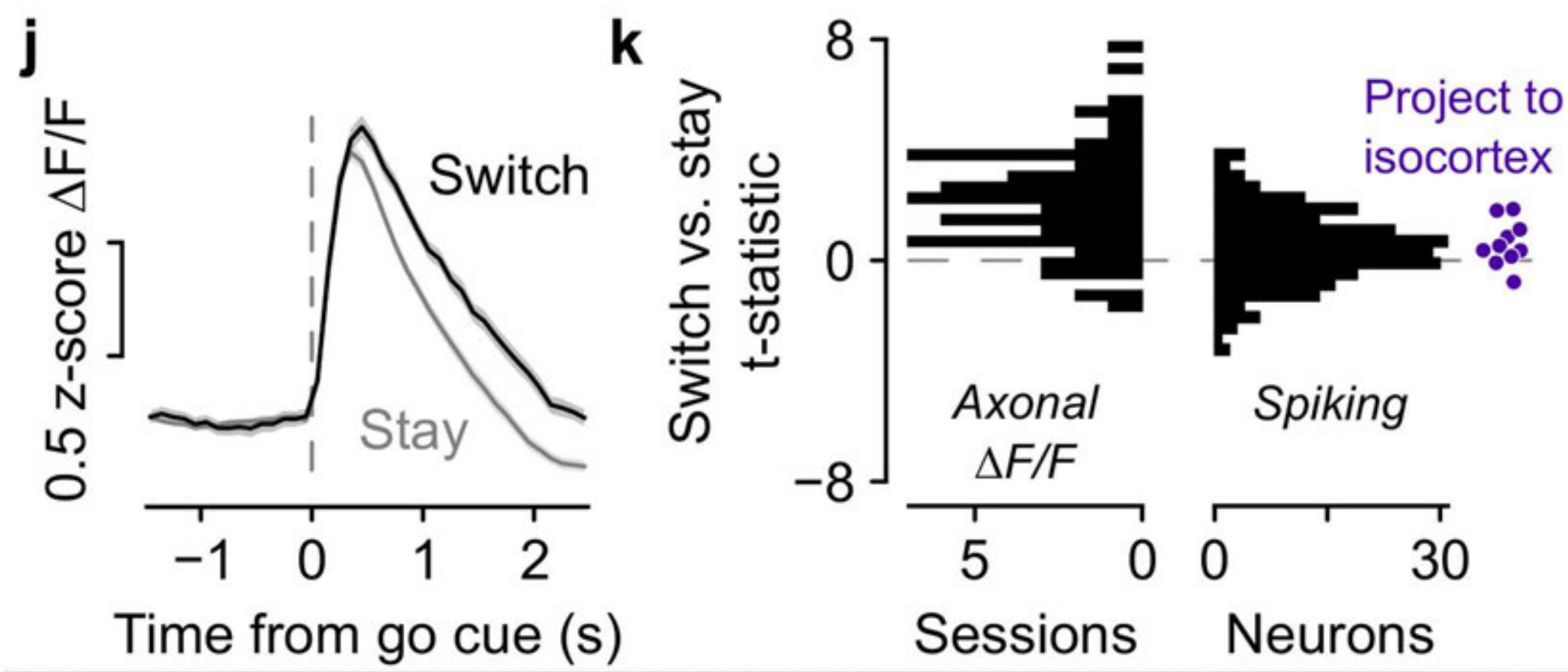

Related to RPE in particular, the new analyses in Figure 6h (below) shows the relationship between LC-NE spiking and trial-by-trial changes in choice behavior. The trial-by-trial changes in behavior correlated in opposing ways with LC-NE neurons that were excited by reward or by no reward:

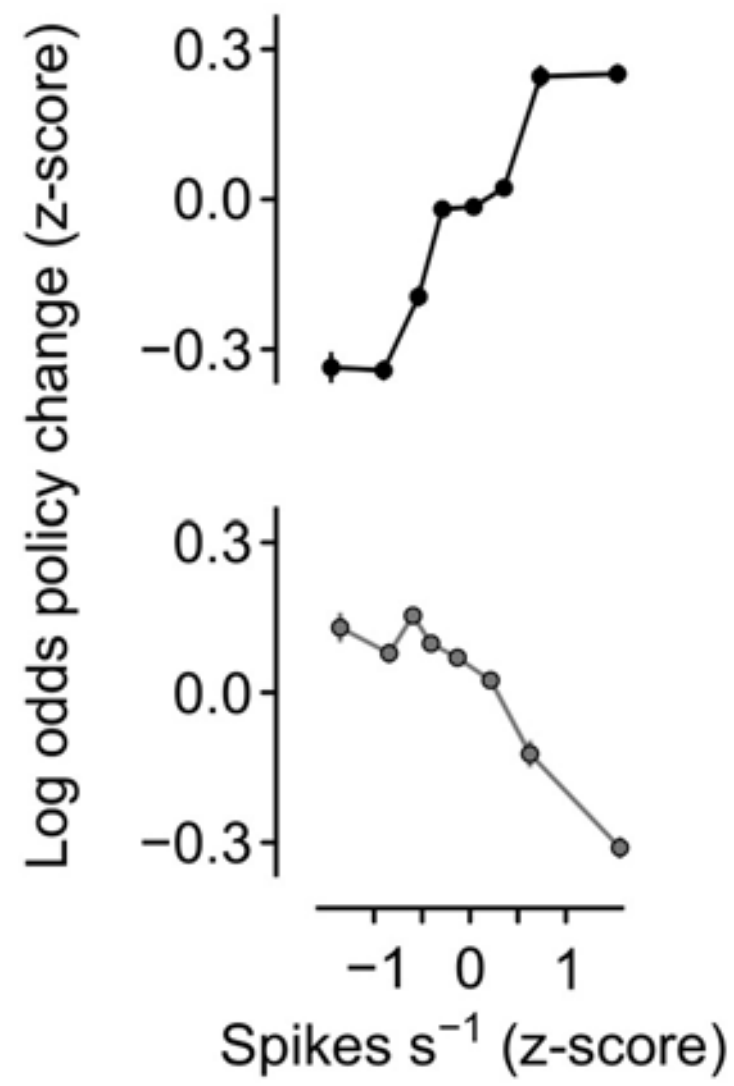

We also analyzed licking movements in more detail and did not find that the fine structure of movements explained RPE correlations.

> *-- Motor versus. cognitive aspects of LC. What is the relationship of LC and licking state? I think it's clear that LC is not just a motor signal but would be great to have a sentence or two with stats saying it has no relationship to licking at baseline. Similarly, what is the relationship of pupils and LC activity (e.g., at baseline)? And, relationship of pupil vs RPE? Does perturbation of LC modulate pupils as strongly during any epoch of the task, or most strongly during outcome?*

We collected new data with high-speed video of the mouse's tongue. We tracked the tongue movements and studied their relationship with choice behavior. These analyses are reported in Figure S12. In Figure S15, we now report the link between LC-NE spiking and licking during trials vs. between trials. There were small effects of licking on LC-NE spiking following the go cue (Figures S15g – S15n). We also compared LC-NE spiking around licks following go cues vs. those during intertrial intervals. We found that LC-NE neurons had larger spike rates during licks within vs. outside of trials, in agreement with R1's assessment that "LC is not just a motor signal."

We added a new supplementary figure, reporting correlations between LC-NE spiking and pupil diameter changes during the task (Figure S16). Overall, correlations were stronger over slow timescales than within trials. There was interesting structure in the pupil diameter changes that did not correlate with LC-NE activity (e.g., dilation during switch vs. stay choices, and variation in pupil dilation with RPE). Although pupil dynamics is not the focus of the present manuscript, the results in Figure S16 will be of interest to investigators who try to use pupil changes to infer LC-NE activity. As noted in response to Reviewer 2 below, we removed the silencing experiment to focus on the new discoveries.

> *(2) The lack of bidirectionality in the LC RPE of the Type 1 cells should be thought about in this report. In the average it appears there is a little activation for all RPEs (positive and negative; Figure 3D). This type of activity that is correlated with RPE may have different functions from a bidirectional VTA dopamine RPE. I would like the authors to*

discuss this in the context of their perturbation, modeling, task, and relationship to other modulators. This issue may also become important as investigators study the functions of Type1 and Type 2 neurons in future studies using aversive stimuli, and in considering how Type 1 cells modulate cortex (e.g., gain versus something else).

This is an interesting point, especially given that's it is unknown whether decreases in LC-NE activity may be seen as analogous to dopamine "dips" that have been proposed to serve as negative RPEs. It's worth noting that we now see a clear monotonic relationship between LC-NE input to frontal cortex and RPE (Figures 6i-6l, shown below). Thus, it's possible that LC-NE "dips" are also relevant for downstream functions.

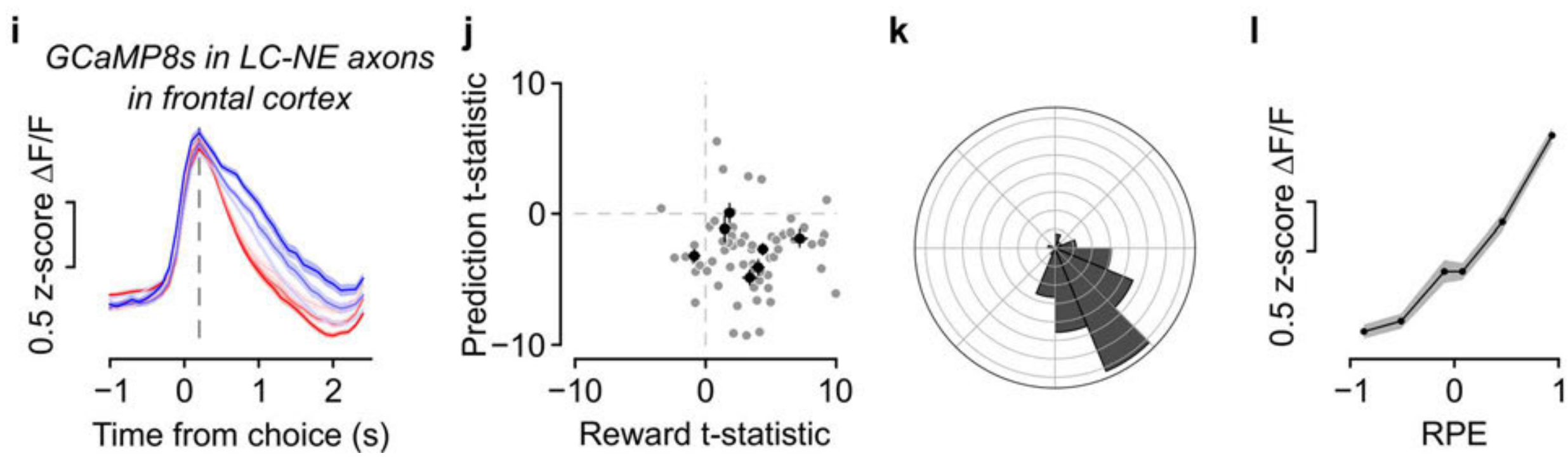

(3) Type 2 neurons did not encode quantitative model derived RPE but did have a strong bidirectional discrimination of + and – RPEs. I found that result very impressive and surprising: as if they are categorically parsing good and bad surprises (even in a bidirectional manner, unlike Type 1). This type of signal could be used in foraging to decide when to go versus stay and for other purposes. I would recommend the authors to discuss this signal more in the Discussion and importantly to acknowledge that Type 2 lack of negative RPE coding maybe due to some complex process such as how they subjectively weight different RPEs or other algorithm differences between Type 1 and Type 2 cells.

Thanks for the suggestion. Our new experiments and analyses show that neurons excited by lack of reward tended to be in ventral/posterior LC (Figure 6a) and showed a negative relationship with future choices updates (Figure 6h). We revised the discussion to speculate about how synaptic inputs and intrinsic properties of LC-NE neurons may produce the different activity patterns we measured.

Also, were Type 2 cells inhibited by Go-cue? Did the Type-1 excitation to go cue have anything to do with trial behavior?

Nearly all neurons were excited by the go cue (Figure 4g, below):

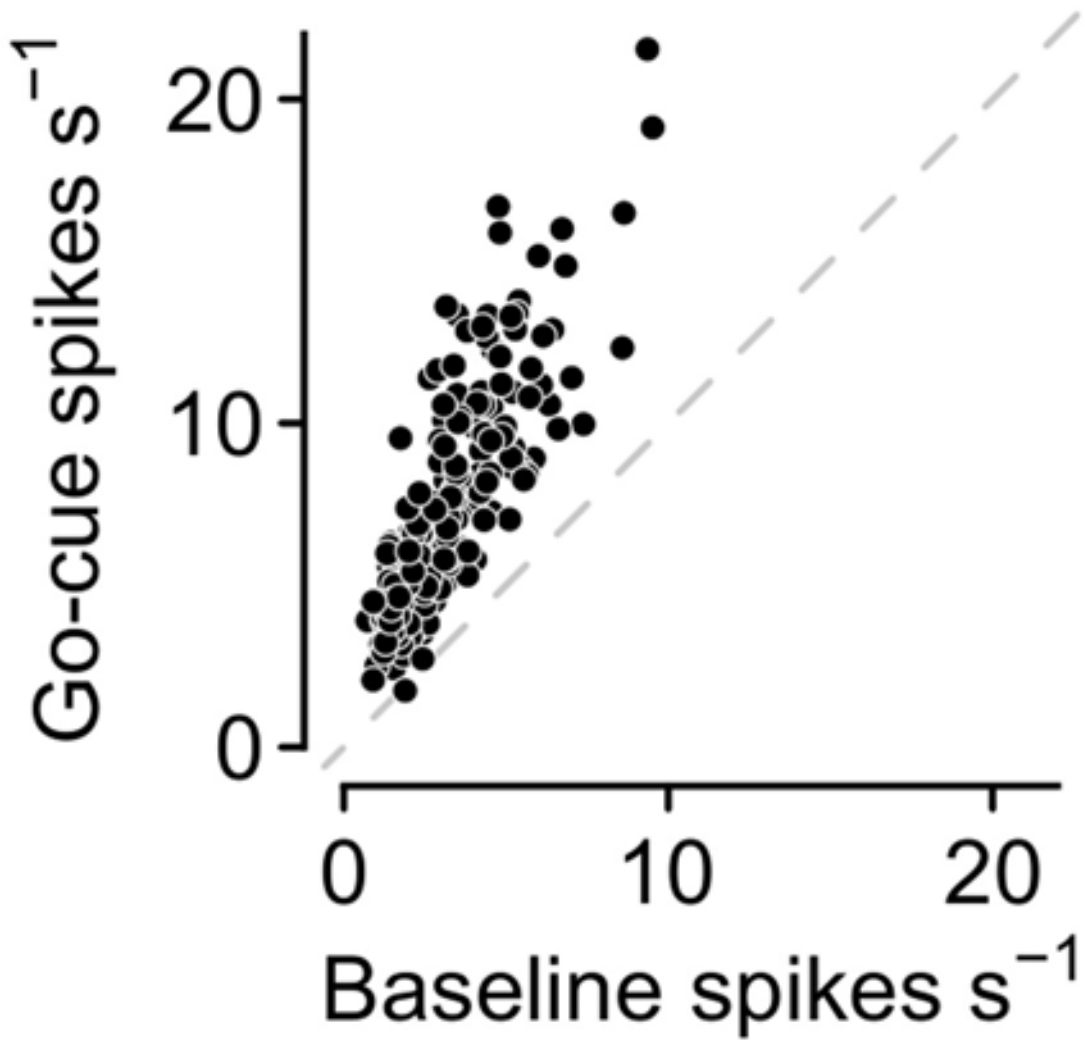

As noted above, the activity following the go cue turned out to relate to the choice (switch vs. stay). This is an important link in the new manuscript between the spatial distribution of axons, genes, and activity.

> *Minor points*
>
> *Maximum magnitude of licking is used to show the degree to which the mice believed they would get a reward in no-reward trials. Why not use licking-persistence also: the duration of significantly elevated licking. This approach works for neural activity (see Zhang et al., 2019) and may work here too.*

Thanks for the suggestion. We added new analyses of lick statistics in the supplement, including lick latency (Figures S12 and S15).

> *Finally, if it was me, I would make some index or measure of behavioral-prediction and compare Type 1 and Type 2 neurons. E.g., how good are switch predictions (or RT predictions in next trial) based on current trial outcome related activity of Type 1 versus Type 2 neurons. After all the complex analyses, that would show readers something simple to "take-away".*

Thanks for this suggestion. As noted above, it led to a new discovery about the relationship between LC-NE activity in different parts of the LC and switch vs. stay choices in this task. This is summarized in Figure 5, with supplemental details in Figure S15.

> *Dayan and Yu, 2006 is cited for determining LC functions in the introduction. I would highlight their contribution by having a separate sentence for biological data references versus modeling references, so that their contribution (modeling) can be appreciated, and so that the readers that are not familiar with the literature can easily understand which previous work was experimental and which was theoretical.*

Along those lines, I would recommend having a few sentences in Discussion dedicated to comparing LC results to SNc/VTA and basal forebrain. I think that would again highlight the importance and uniqueness of the current work.

Given the substantial reworking of the manuscript, we focused discussion on LC, with a brief comparison to dopamine neurons in the Discussion.

Reviewer 2 (R2)

We thank R2 for their insightful input on the previous version of the manuscript. Their main points focused on concerns about the silencing experiments, as well as the spatial distribution of activity of different neurons. The latter spawned a substantial update to the manuscript, including the discovery of a spatial organization in LC.

> *The manuscript by Su & Cohen attempts to address an important question in the field relating to how norepinephrine release in the cortex influences learning, and whether modularity exists at the level of the LC which may affect trial-by-trial learning. This is currently an actively debated topic with respect to the LC, it's larger roles in behavior, and the actions of norepinephrine generally. So the authors do test a novel, important and impactful series of hypotheses in this study. The studies is rigorously designed, modeled, and conducted overall, however there are concerns about the strength of the conclusions as it currently stands.*
>
> *Using optogenetic tagging of norepinephrine-producing LC neurons (Dbh+ LC-NE neurons), Su & Cohen record from many LC-NE neurons (149 neurons from 4 mice) simultaneously in an awake behaving mouse performing a complex reinforcement learning task. Consistent with previous work, they identify two main classes of LC-NE neurons based on their electrophysiological properties, type I neurons, characterized by a wide action potential shape, and type II neurons with thinner action potential waveforms. While these classes were known, examining their role in reinforcement learning is novel and quite interesting a finding. The authors demonstrate differences between the two classes and reinforcement learning measures, primarily that the response of type I neurons is graded and scales with RPE, while type II LC-NE neurons are excited when reward is expected but not delivered (negative RPE). These findings are novel and extremely interesting, and warrant deeper analysis.*

Thanks for the enthusiasm and push to do deeper analysis to better understand the projection specificity. We added substantial new data that led to a new understanding of the spatial organization of LC-NE activity (and anatomy).

We studied the anatomical organization of LC-NE neurons. We found a strong spatial organization of both the axonal projections (Figures 1, 2, and their supplements) and gene expression (Figure 3). These findings inspired us to better understand the spatial distribution of cellular activity in the behavioral task.

Thus, we doubled the sample size of single units, with data from Neuropixels probes. We performed antidromic stimulation over the cortex and, using collision tests, identified a sample of neurons with projections to frontal cortex. We localized the cells in the LC in standardized mouse brain coordinates, using a new atlas we generated for this study (Figures 1a, 1b, and S1). As a result, we were able to analyze the spatial distribution of single neuron activity. We found a clear spatial relationship between activity related to task engagement (Figures 5a-5d and S15) and switch vs. stay choices (Figures 5e-5h and S15). Single cells and bulk projections to frontal cortex selectively correlated with RPE (Figure 6).

> *Unfortunately, the subsequent functional experiments manipulating type I neurons optogenetically and assessing the activity of LC-NE projections during reinforcement learning suffer from numerous issues leaving their interpretation challenging, and tempering enthusiasm for the manuscript as a whole. The authors' claims about LC-NE*

influencing RPE can be further strengthened and the noradrenergic RPE signal at the frontal cortex can be further clarified.

To focus on the spatial organization of both anatomy and physiology, we removed the experiments silencing activity during behavior. Note that we now find a gradation in LC-NE spike waveforms, consistent with our observations of graded projections and gene expression.

Major comments:
1) The authors use optogenetic inhibition of LC-NE neurons via the excitatory opsin GtACR2, and attempt to preferentially inhibit type I neurons, which should reduce RPE on stimulation trials and decrease the value of the selected action. However, no evidence for preferential targeting of type I neurons is really presented. This is somewhat problematic because the opposite prediction would be made if type II neurons are inhibited. While the authors state "low" irradiance is used (still in the mW range), there is no data nor histology presented to fully support this contention. The authors may wish to try strategies using tapered or fibers/LEDs that direct light at an angle, or potentially present histology demonstrating bleaching selectively of the fluorophore in dorsal LC-NE neurons. As it stands, given that type II neurons are 1) more prevalent overall in the LC than type I neurons (86 out of 149 LC-NE neurons were type II), 2) have higher baseline firing rates than type I neurons (Extended Data Figure 2c), and that the only histological image of GtACR2 expression shows high expression in both dorsal and ventral LC (Figure 4a), it is hard to justify a hypothesis and subsequent predictions based on manipulation of type I neurons only. This is the biggest concern related to the conclusions of the paper. If this manipulation is the effect of inhibiting both type I and type II neurons, the relevance of their RPE-specific activity patterns becomes unfortunately more tenuous and less novel.

Our new results on the structure of LC and the spatial distribution of activity related to choice (Figure 5) and reward (Figure 6) highlight the complexity of this experiment. In particular, we show LC-NE neurons form a single distribution of a cell type (Figure 3), projections (Figure 2), and activity (Figures 5 and 6). Thus, as noted above, we removed this figure.

2) A similar question exists with the following experiment using fiber photometry to examine LC-NE neuron activity as well as the activity of LC-NE terminals in the medial PFC. At the somatic level, it is unclear why photometry signals preferentially resemble type I and not type II neurons. Again, a careful attempt to quantify and present histological targeting of the fibers within the LC would be very useful. If fibers were dorsal, a strong type I signal might be expected. Given the extremely low N in this experiment (n = 2), there is much to be gained from recording additional mice and correlating signals with location of the photometry fiber in the D/V axis. If a photometry signal obtained in very ventral LC (where type II neurons are prominent) does not resemble the convolved signal found in Figure 5c, then interpreting a lack of this signal in the PrL may be more spurious. Overall, more experiments in this case are needed to draw firmer conclusions about the nature of the signal in both LC and PrL. The authors have the ability to do this, so it is more about adding N's than technically challenging an approach.

Thanks for this suggestion. Given the clear spatial organization of single-cell activity, we removed the bulk measurement from the somata to focus on the spatial organization across the population, and the specificity of the activity in the cortical projections (see response to question 6 below).

3) In the silencing of type I LC-NE neurons, the authors predicted that it will lead to artificially reducing RPE, and therefore decreasing the value of the chosen action and increasing the P(switch). Yet, in Fig 3f, the authors showed that "Higher activity of type I neurons correlated with larger change in choice likelihood". Thus, it would be particularly helpful if the authors can clarify the discrepancy between their observation and the model prediction. As stated above a weakness in the silencing experiment is the assumption that type I neurons are preferentially activated using the low irradiance by targeting the more dorsal neurons. From Fig 2f, the type II neurons appear to be ~20% of the neurons in the dorsal layer, thus, their participation in RPE cannot be discounted, especially with relatively high correlation of choice switching at its lower firing range (Fig 3f). What would incorporating predictions of type II neuron silencing look like? Does the degree of silencing as captured by the change in pupil diameter (Fig 4d) correlate with the probability of switching (Fig 4f)? These are important considerations and more experiments are needed to firm the conclusions at the later end of the manuscript in this regard. If possible to complete the novelty and impact of the findings are most certainly very exciting.

As noted above, we removed this experiment. (Note that the observation of a positive correlation between future choice and higher activity in RPE-correlated cells [Figure 6h] is consistent with the hypothesis that reducing their activity would increase P(switch).)

4) Also, with the GtACR2 silencing, the protocol uses a 2s constant stimulation followed by a 2s ramp down to minimize post-inhibitory rebound excitation, but from the changes in pupil diameter (Fig 4c), the increase right after stopping the laser suggests that a rebound depolarization may still be present and represented in the activity. Furthermore, per the methods section, the trial duration for silencing experiment is 3.0s with a minimum of 0.1s inter-trial intervals, it is unclear whether the silencing duration of 4s is bleeding into the initiation of a new trial. A bit more characterization of the inhibition parameters might be helpful. Perhaps the authors can do a few slice experiments to characterize these parameters, to guide the in vivo work. Of course they aren't totally equivalent, but it might make the other concerns above easier to address if there is a better set of inhibition parameters defined. Alternatively, the authors could label the dorsal neurons via a retro-CAV approach

As noted above, we removed this experiment. (Note that in the previous experiments, the change in pupil diameter was due to the unchosen lick tube returning to its original position, rather than light offset.)

5) Additionally, given the approximate linear relationship between type I neurons and RPE and choice switching, it would further strengthen the authors claim if they also preferentially activated the type I neurons to determine whether their model predictions will be correct.

As noted above, we removed this experiment.

6) In correlating LC-NE activity in the PrL to the RPE, the authors saw that the linear relationship holds at positive RPE, but does not change at negative RPE, and attributed the lack of positive correlation at negative RPE to a difference in measurement technique. While fiber photometry may be less sensitive at axonal terminals, it would be informative to have LC-NE calcium activity measured by fiber photometry at the soma to

see if this may indeed be the explanation. Moreover, a measurement of NE in the PrL during the reinforcement learning assay would lend further support to the specific role of NE in signaling RPE.

We increased the sample size of the LC-NE axonal GCaMP recordings in PL and found a clear monotonic relationship between activity and RPE (Figures 6i-6l).

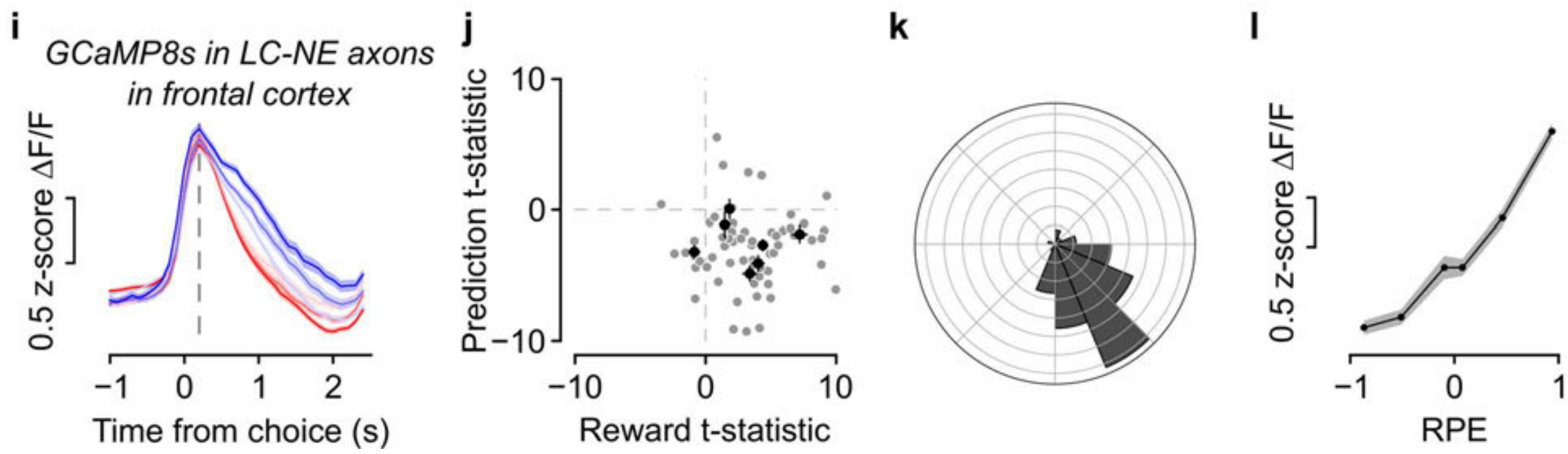

This matches the results from the new data from antidromically-identified neurons with projections to cortex (Figures 6e and 6f). In separate experiments, we measured fluorescence from an NE sensor (GRAB-NE) in PL and found that it also correlated with RPE (see below). We did not include this experiment in the manuscript, as we have some concerns about the sensor's selectivity and wished to focus the manuscript on cellular activity.

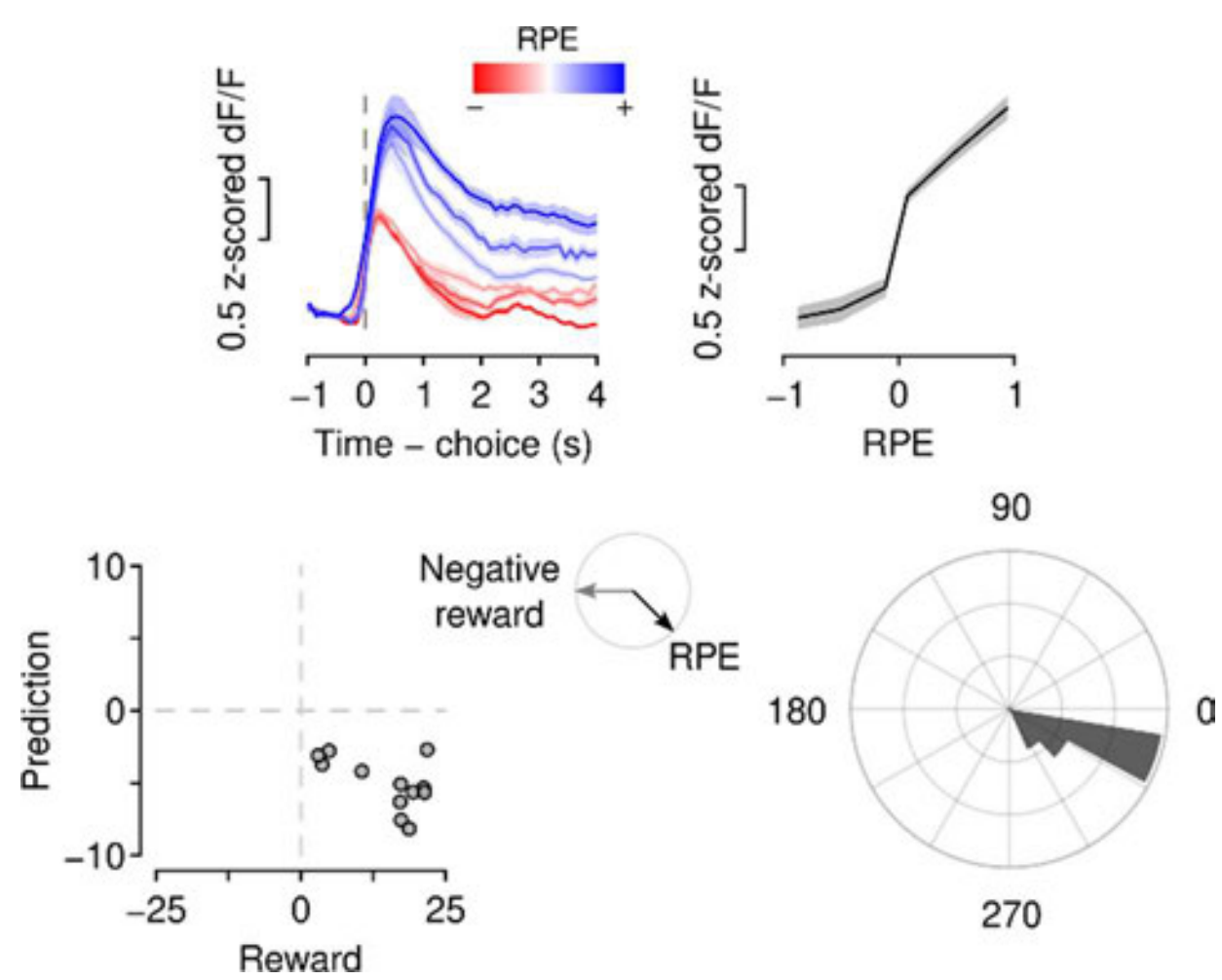

Minor comments:
-The methods state that 7 mice were used for GtACR2 and control experiments, but the data plotted in Figure 4 make it appear that there are more mice in each group. Perhaps I was confused about how this was conducted.

-It is unclear if GtACR2-expression itself is affecting mice behavior, as the mice cohort overall appears to have slower response time than the EYFP cohort (Extended fig 4b) regardless of laser use, and had much higher lick rate in response to reward regardless of laser use (Extended fig 4c).

As noted above, we removed this experiment.

Reviewer 3 (R3)

We thank R3 for their enthusiasm for the first version of the manuscript. They noted the clear results and asked about LC-NE anatomy. This led us to new discoveries that form the foundation of our revised manuscript.

> *The authors report on a study in which they identified, based on waveform shape and anatomical location, two types of NE neurons. They then examined the responses of these neurons, as well as the effects of silencing these populations on choices in a restless bandit RL task. They found that the more dorsal population of type I neurons coded a positive RPE, whereas the more ventral population of type II neurons coded lack of reward. Preferentially silencing type II neurons led to an increase in switch probability following a lack of reward.*
>
> *This is a nicely done study with some novel findings. The behavioral is well-controlled, and the authors convincingly show differences in the cell populations with respect to response properties. The opto inhibition experiments are also interesting.*
>
> *I have two primary comments on this manuscript. First, with respect to the dopamine vs. norepinephrine innervation of cortex, it is not so clear that they differ that much. Perhaps there are clear differences in the rodent in the dopamine vs. norepinephrine innervation of cortex, but I'm not aware of studies showing this. Also, there is not much norepinephrine in the anterior striatum, but I believe there is more in the caudal striatum, and it's possible that the caudal striatum is mediating these effects.*

Thank you for the question about LC-NE anatomy. Prior to our current work, the literature had no information about the complete projections of individual LC-NE neurons, nor their links to molecular variation among the population. Our revised manuscript now includes a comprehensive study of the morphologies of LC-NE neurons, at the population level (Figures 1, S1, and S2) and at the single-cell level (Figures 2 and S3-S7), as well as their links to cell type (Figures 3 and S8-S11).

We found LC-NE projections across the brain and spinal cord, with minimal innervation of much of the striatum (relative to other structures). For example, below is an image from the supplemental movie, showing LC-NE axons labeled in one hemisphere (note extensive innervation of ipsilateral cortex, with few axons in striatum).

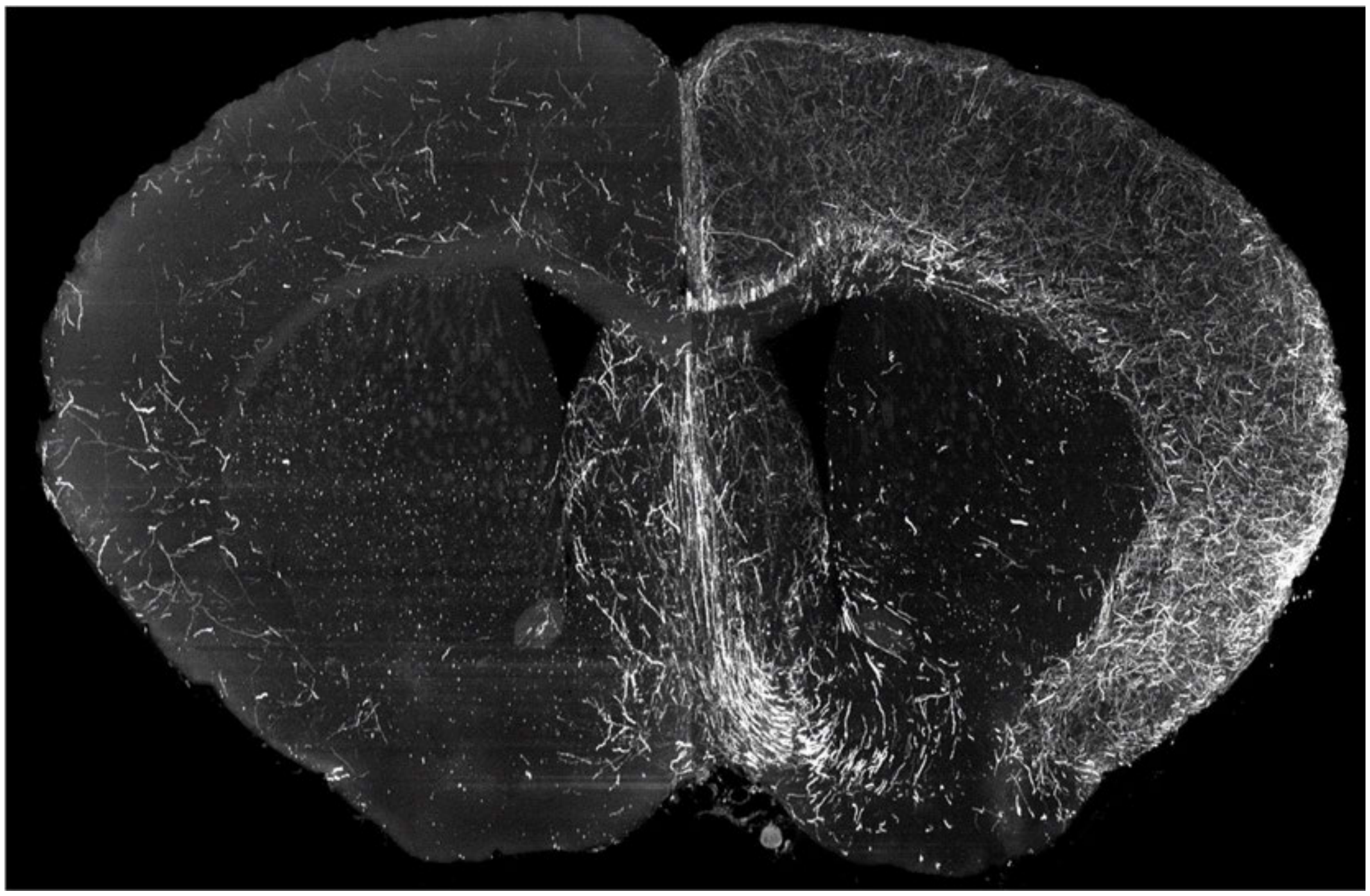

Using new labeling and microscopy techniques, we reconstructed the complete morphologies of 130 LC-NE neurons (Figures 2 and S2). Single neurons had broad projections to subsets of brain regions (e.g., Figure 2b below):

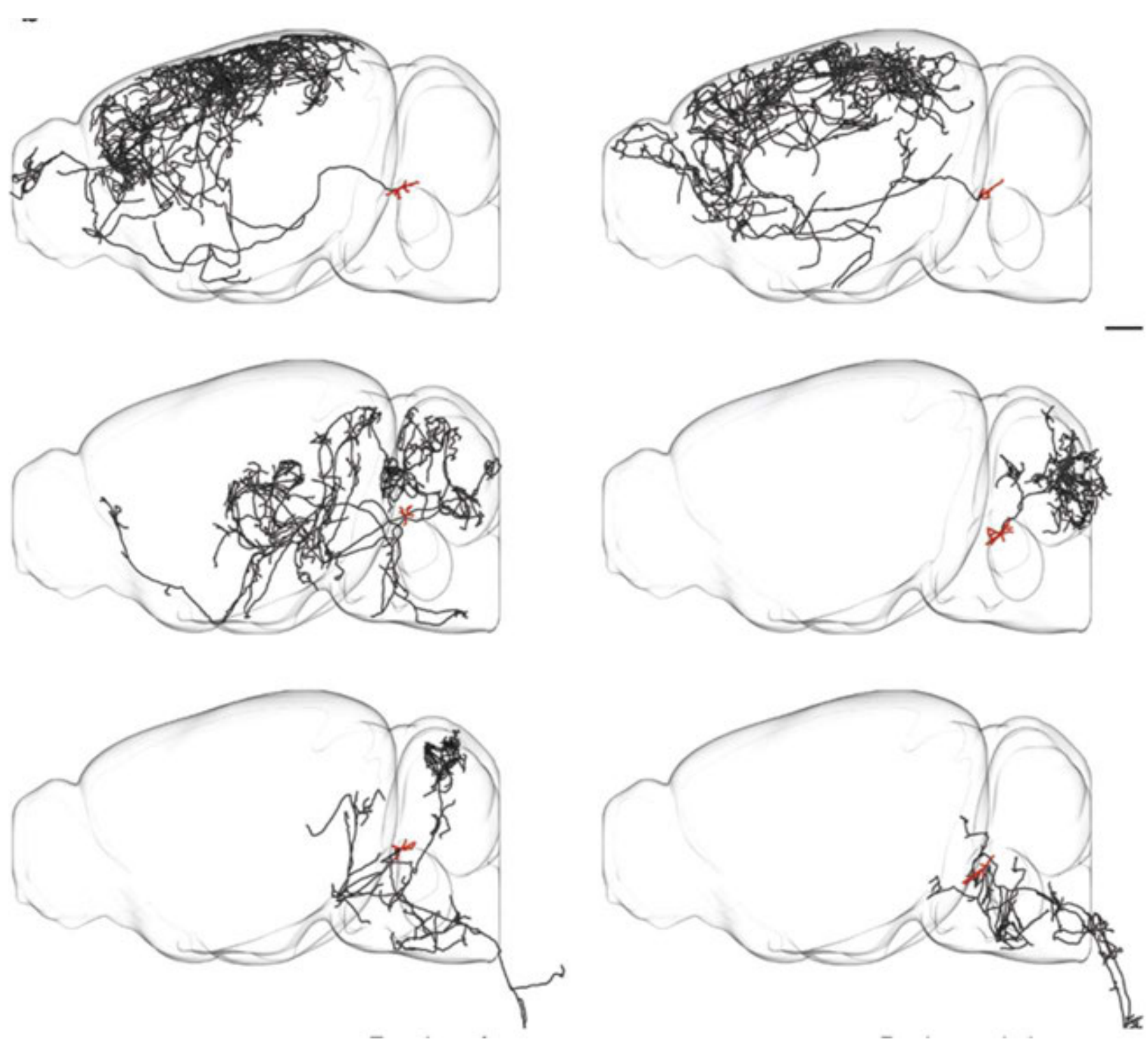

Very few axons were found in striatum (included in cerebral nuclei, CNU; Figure 2c):

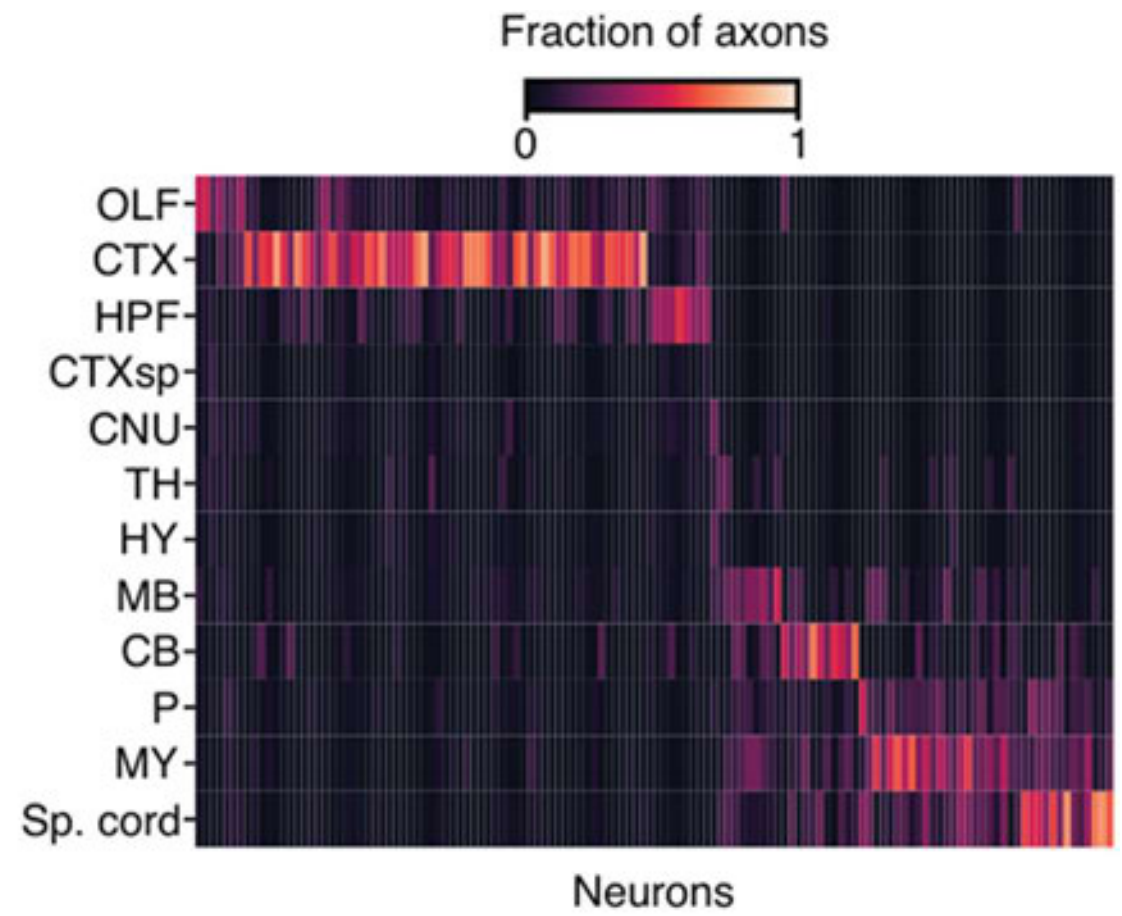

Remarkably, LC-NE neurons innervating different regions of the brain and spinal cord had cell bodies distributed across the dorsal-ventral axis of LC (Figure 2d):

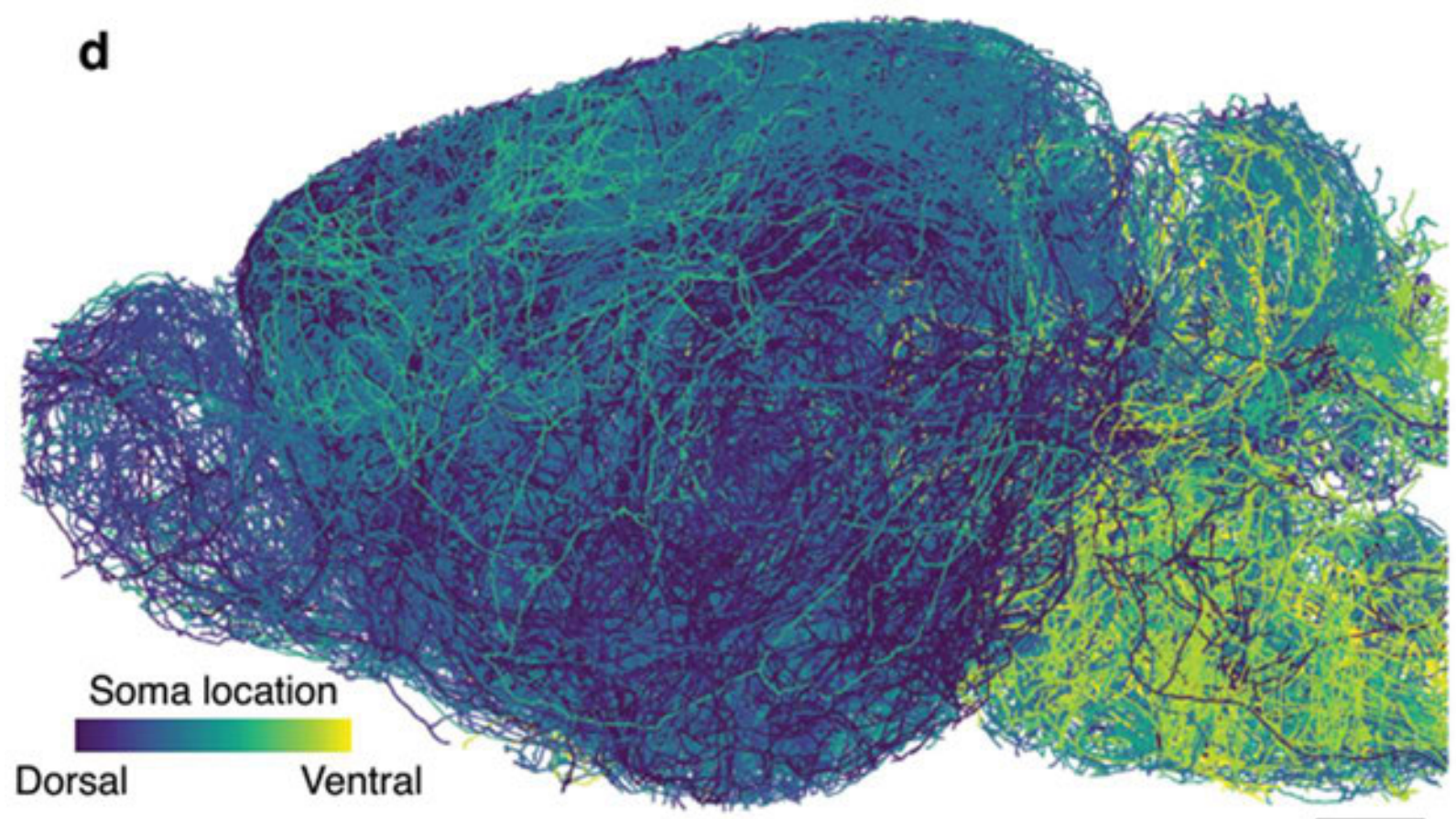

Although we did not study dopamine neuron anatomy here, the ventral midbrain dopamine innervation of striatum and NE innervation of the mouse isocortex appear quite complementary to that of LC (Nomura et al., 2014, reproduced below).

[Redaction] Includes Third Party Material

Figure 1 from Nomura et al., *Front Cell Neurosci*, 2014

In macaques, there appears to be denser dopamine neuron innervation of isocortex than in rats or mice, but here, too, there are regions with very sparse dopamine input (e.g., V1; Berger et al., J Comp Neurol 273, 99, 1988). Overall, we agree that regional differences in dopamine vs. NE innervation of different parts of cortex and striatum deserve further study but are outside the scope of this manuscript.

> *Second, while the experiments nicely show differences in the directional licking task, there are now numerous papers showing that, while the responses of dopamine neurons and the effects of their manipulation in similar tasks might be clear, the functional role of dopamine neurons is actually far more complex. Thus, the results in this simple experiment on NE are highly interpretable. But it seems that subsequent experiments with more complex paradigms would likely lead to different interpretations.*

We agree that the results are highly interpretable and, indeed, view it as a strength of the study. It will be important to study LC-NE neurons in multiple behavioral tasks in the future to determine whether our observations generalize to other behaviors.

> *In sum, while this is a nice study, I think a more convincing case would require the use of multiple paradigms looking at factors like sensory preconditioning, outcome type switching, perhaps motor behavior, the explore-exploit tradeoff (and arguably other factors too numerous to list), along with a direct comparison of the effects of dopamine manipulations in matched experiments. This, combined with a clear anatomical study of projection patterns of dopamine vs. norepinephrine, and maybe information on which targets are most relevant for dopamine vs. norepinephrine in particular tasks.*

We agree that recent literature on the heterogeneous role of dopamine neurons in reinforcement learning serves as a nice reminder that our results should not be overinterpreted. We changed much of the text to reflect this (including the title), focusing on the link between structure and function. We hypothesize that LC-NE neurons may play a role in learning and

leave it to future studies to measure their activity in different behaviors, including tasks that can access explore-exploit tradeoff, and a comparison with dopamine neuron activity (not the focus of this study). Note that in the present behavioral task, we made three key observations about LC-NE activity in different time windows: (1) the relationship between background activity in the intertrial interval and task engagement; (2) the relationship between activity at the beginning of the trial and switch vs. stay choices; and (3) responses to the outcome (presence or absence of reward). Whether these observations generalize to other behaviors will be an important subject of future research.

ALLEN INSTITUTE for NEURAL DYNAMICS

Jeremiah Y. Cohen, Ph.D.
Allen Institute for Neural Dynamics
Seattle, WA

July 21, 2026

Dear editors,

We thank you and the reviewers for the constructive and positive feedback on our previous submission. Below are the updates to the manuscript, in response to reviewer comments.

For the authors,

Jeremiah Cohen

Jeremiah Y. Cohen, Ph.D.

Reviewer 1

> *Thank you for addressing all of my questions and concerns. This is a remarkable and elegant piece of work.*

Thanks for your enthusiasm for our study.

Reviewer 2

> *In this substantially revised manuscript from the Cohen group and Allen Institute, the authors have now provided one of the first and most comprehensive data sets on LC heterogeneity across both molecular and anatomical domains, which is then further bolstered by behavioral data and electrophysiology demonstrating functional separation between the dorsal and ventral LC populations with meaningful implications for understanding the LC-NE system in learning and other behavioral domains. The paper is a tour - de - force, and will become the new reference atlas for LC circuits going forward. I only have a few minor corrections to suggestion before the MS is acceptable for publication.*

Thanks for your enthusiasm for our study.

> *The main figures of the paper tell a very strong narrative regarding the anatomical separation of LC, across various domains, but what is sort of missing, but buried in the supplement is the molecular profiling and BARseq data. It would be useful to remove some of the anatomy, or simply add a few panels which include more of the merFISH or BARseq data in the main manuscript.*

Thanks for the suggestion. We added panels to the Extended Data to expand the analysis of gene expression. We chose genes based on recent studies of LC-NE transcripts: Hughes et al. (2024) and Luskin et al. (2025). This covers a more extensive set of genes that we hope will be useful for generating hypotheses for future studies of LC-NE neurons (e.g., genes that regulate growth or activity of LC-NE innervation of different CNS regions).

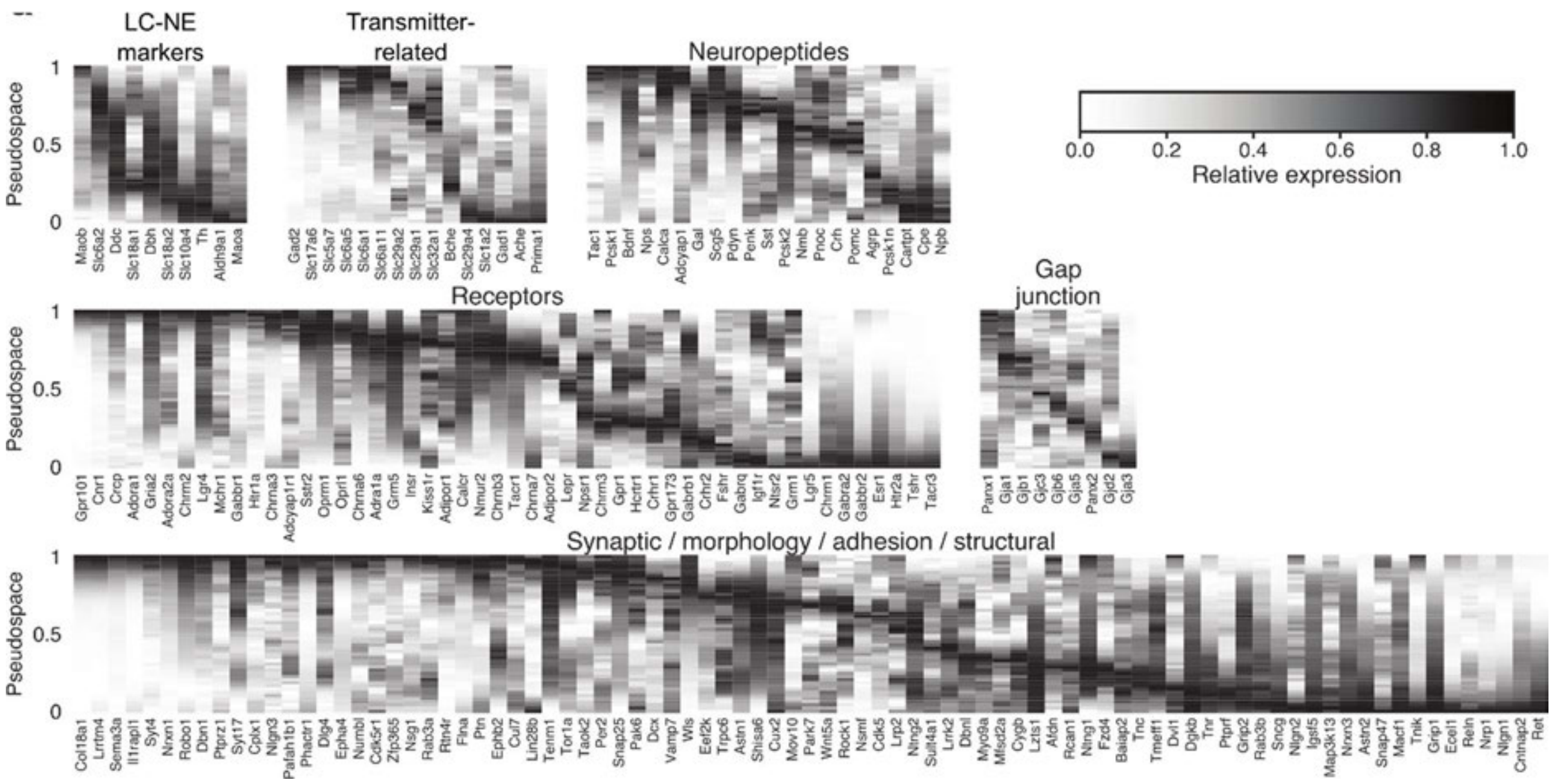

On a related note, it is difficult in the current form to access the seq data in terms of looking at other expression of other genes not currently shown in the figures or analysis? It would be useful to see data sets from the seq data that compare to smaller data sets and the Allen Cell Atlas in terms of expression profiles for genes, know to have -cre driver line access, so that the field can potentially independently synthesize combinations of expression patterns with cre- and/or flp lines for accessing some of the unique LC projection modules highlighted here. Maybe I missed where that data is, but since there are 3 other LC seq data sets, some comparisons or mention to some of the genes IDed in those data sets, or the lack thereof, could be useful for integrating this nice body of work with that larger field. For me, this is simply show more plots (dot plots or other they have used) for other genes in the LC which are relevant. The data as presented in MERFISH (it isn't clear if this was a custom run, or a run only using the baseline MERFISH gene set), so it would just integrate this new work more into that existing framework. Albeit, the other data sets have a lot less read depth and cells, but since many of the populations overlap here, I think a little more effort to share more of that data is warranted in the final main figures of the MS. S9 and S10 contain some of this information, but there is pharmacological evidence that there are differences in neuronal responses to various receptor ligands in LC, and so a little more of what was looked for in that domain could be presented in some form, unless those genes weren't assessed by the MERFISH.

In addition to the new Extended Data figure panels (above), and updated MERFISH panels in Extended Data Figure 10, all data are publicly available for the community to analyze further. We hope these data will be useful for other investigators!

Reviewer 3

I would like to thank the authors for revising the manuscript and replying to my initial comments. The study provides descriptive information about the anatomy of the LC and its projections, and the activity of the neurons in one task. While I appreciate that the authors would like to limit the scope of their paper, I am not sure that I find that the

results provide a clear understanding of either the anatomy or the function of the LC. Furthermore, a contrast with other systems would be very useful, but it is not provided.

We are excited about our new understanding of both the structure and function of LC-NE neurons. We acknowledge that a deeper understanding will require measurements of activity in this system during many behaviors, as well as a thorough characterization of morphologies, gene expression, and activity in other neuromodulatory systems. We hope that our work stimulates future research on these and other exciting directions.
